# Patient’s assessment and prediction of recovery after stroke: a roadmap for clinicians

**DOI:** 10.1186/s40945-023-00167-4

**Published:** 2023-06-19

**Authors:** Silvia Salvalaggio, Leonardo Boccuni, Andrea Turolla

**Affiliations:** 1 Laboratory of Computational Neuroimaging, IRCCS San Camillo Hospital, Venezia, Italy; 2 Padova Neuroscience Center, Università Degli Studi Di Padova, Via Orus 2/B, 35131 Padova, Italy; 3 Institut Guttmann, Institut Universitari de Neurorehabilitació Adscrit a La UAB, Badalona, Barcelona Spain; 4 Universitat Autònoma de Barcelona, Bellaterra (Cerdanyola del Vallès), Spain; 5 Fundació Institut d’Investigació en Ciències de La Salut Germans Trias I Pujol, Badalona, Barcelona Spain; 6 Department of Biomedical and Neuromotor Sciences (DIBINEM), Alma Mater University of Bologna, Via Massarenti, 9, 40138 Bologna, Italy; 7 Unit of Occupational Medicine, IRCCS Azienda Ospedaliero-Universitaria Di Bologna, Bologna, Italy

**Keywords:** Prognosis, Prediction, Clinical-guide, Motor recovery, Stroke, Neurological physiotherapy, Assessment

## Abstract

**Background and purpose:**

In neurorehabilitation clinical practice, assessment is usually more oriented to evaluate patient’s present status, than to plan interventions according to predicted outcomes. Therefore, we conducted an extensive review of current prognostic models available in the literature for recovery prediction of many functions and constructs, after stroke. We reported results in the form of a practical guide for clinicians, with the aim of promoting the culture of early clinical assessment for patient stratification, according to expected outcome.

**Summary of key points:**

To define a roadmap for clinicians, a stepwise sequence of five actions has been developed, from collecting information of past medical history to the adoption of validated prediction tools. Furthermore, a clinically-oriented organization of available prediction tools for recovery after stroke have been proposed for motor, language, physiological and independency functions. Finally, biomarkers and online resources with prognostic value have been reviewed, to give the most updated state of the art on prediction tools after stroke.

**Recommendations for clinical practice:**

Clinical assessment should be directed both towards the objective evaluation of the present health status, and to the prediction of expected recovery. The use of specific outcome measures with predictive value is recommended to help clinicians with the definition of sound therapeutic goals.

## Introduction

Predicting events in medicine is fundamental for giving clinicians, patients and caregivers answers regarding what is likely to be expected for their clinical conditions in the future [1, 2], thus to plan the best therapeutic approaches accordingly. The first prognostic model was developed in 1953 for patients with myocardial infarction, introducing the concept of quantifying estimate of risk mortality and life expectancy [3]. To date, most of the neurological literature has been focused on the study of spontaneous recovery, thus *Prognosis.* Rehabilitation, however, has the role of spreading the concept of *Prediction*, thus considering the rehabilitation intervention as a proper driver factor of recovery, influencing prognosis. This terminological difference is confounding and makes its use misleading, as the application of a prognostic model in the rehabilitation field presupposes that the rehabilitation intervention is actually present, which could change the expected outcome. Therefore, in order to overcome this problem, in this paper we use the term *Prognosis* only in reference to prognostic models, and the term *Prediction* for the concept of predicting recovery as the expected outcome of a rehabilitation pathway.

Indeed, physical therapy refers to the forecast of optimal level of functional improvement to be expected in a certain time frame while performing some kind of rehabilitative intervention. Moreover, prediction of recovery potential may be used as guidance for setting concrete goals with patients, thus offering patients tailored rehabilitation program according to their potential, and finally for monitoring and interpreting patient’s achievement over time [4]. However, prediction of recovery is not applied systematically in rehabilitation settings, leading to unawareness of potentials of recovery for both clinicians and patients. For instance, a recent survey shown that only 9% of physiotherapists and occupational therapists use prognostic tools in clinical practice, despite the vast majority (89%) of them acknowledge their importance for predicting recovery potential, after stroke [5].

The first step of prediction relies on proper assessment; therefore, clinicians should dedicate sufficient time and resources for developing comprehensive clinical assessment strategies. In the field of neurological rehabilitation, a patient-centered integrated framework for decision making was proposed, that considers assessment, diagnosis, prognosis and plan of care as a circular pattern of patient care [6]. In this perspective, being familiar with interpreting initial signs and symptoms, selecting the most appropriate assessment strategy and using prediction models is pivotal to be time and clinically efficient. However, referring to evidence for each step of the process requires significant knowledge of the available literature, which is not always feasible for clinicians deploying daily rehabilitation services.

The aim of the present article is to provide readers with an update on reliable strategies to predict potential of recovery after stroke. Therefore, we propose a pragmatic and user-friendly guide for clinical examination and decision, based on outcome measures and strategies with some evidence from literature of their utility in recovery prediction.

### Clinical steps towards prediction of motor recovery

**Fig. 1:**
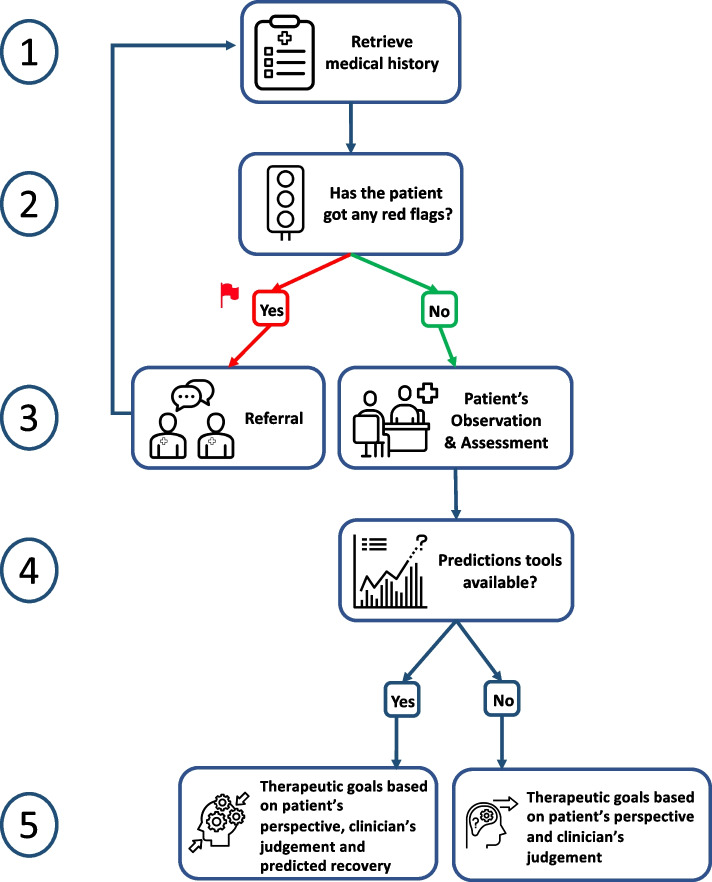
Five steps towards the definition of therapeutic goals, from medical history to the use of recovery prediction tools

Recovery prediction requires a stepwise approach for correct application of available tools, whose actions can be summarized into five steps: (i) collection of relevant information from medical history, before first meeting with the patient; (ii) exclusion of critical clinical conditions requiring referral to other specialists (indeed, steps i and ii are iterative throughout the whole period in which the physiotherapist has the patient in charge); (iii) patient assessment during the first meeting, for quantification of impairments (body function and structure) and restrictions (activity and participation); (iv) framing prediction of recovery by using prognostic tools whenever available; (v) definition of rehabilitation (shared) goals and therapeutic interventions (Fig. 1).

#### Step 1. Collect patient’s relevant information from medical history

Before meeting the patient, collecting information is fundamental by reading medical records and receiving feedback from medical doctors and other healthcare professionals that already got in contact with the patient. Important information to be retrieved are:

- Demographic characteristics: age, sex, nationality, native language.
- Main diagnosis: medical-clinical condition, date, circumstances, aetiology and physiopathology of stroke event (ischaemic, haemorrhagic, size, localization), presence of medical complications, and whether the patient is clinically stable.
- Medical history: presence of other disease, risk factors (e.g. atrial fibrillation, high blood pressure, diabetes, smoking, high blood cholesterol levels), ongoing drug intake, previous rehabilitation interventions (what, where, when and how long):
  - o Previous medical examinations.
  - o Presence of imaging records (e.g. MRI, CT) or neurophysiological (e.g. TMS, EEG, EMG) exams, evaluating specific features of stroke diagnosis (e.g. presence of Motor Evoked Potentials, level of asymmetry of fractional anisotropy in the white matter).
- Complementary personal information: work and employment, social environment, hobbies before stroke.

This information has to be considered as useful, but not mandatory, since the clinician is allowed to require them subsequently if necessary.

#### Step 2. Evaluation of potentially critical clinical conditions

Before meeting with the patient, any critical clinical condition should be detected and referred to the competent specialist. Beyond that, presence of any critical sign or symptom must be detected by the physiotherapist as long as the patient is in charge, in a rather iterative way along the course of the treatment. Stroke sequelae may involve different domains (e.g. cognitive, language, motor), therefore team-work with other healthcare providers (e.g. speech language therapist, neuropsychologist) may be necessary for a more comprehensive care of the patient.

#### Step 3. First meeting: observation and assessment

The first meeting with the patient is fundamental to establish a trust-based therapeutic alliance, also considering the environmental setting (e.g. hospital room, intensive care unit, gym). In addition to that, detection of stroke severity should be determined based on:

- *Level of consciousness (wakefulness, awareness and interaction).* Can be assessed by observation (e.g. patient awakening, eyes opening) and reaction to simple requests (name telling, moving limbs), or using outcome measures such as:
  - o Glasgow Coma Scale (GCS) [7]
  - o National Institutes of Health Stroke Scale (NIHSS) [8] – subitem 1a, 1b, 1c;
- *Cognitive status, language and communication barriers.* These aspects can be qualitatively assessed during the previous step, by evaluating awareness of what is happening in the surroundings and analysing answers to simple questions. Alternatively, the most indicated outcome measures in this phase are:
  - o NIHSS [8] – subitem 9, 10
  - o Western Aphasia Battery [9]
- *Evidence of neurological deficits (e.g. cranial nerve impairments, extinction phenomena, inattention, anosognosia).* The most appropriate method for the evaluation of these neurological aspects is performing a neurological examination (II, III and VII cranial nerves) or using a validated outcome measure, such as the NIHSS [8] – subitem 2, 3, 4, 11.
- *Evaluation of trunk control, ability of rolling and sitting.* Assess these patient’s abilities by observation or use of validated outcome measures, such as the Trunk Control Test (TCT) [10].
- *Assessment of standing balance and ambulation.* Examine the patient during transfers from chair to bed, from sitting to standing position, and observe movement pattern during walking. The following outcome measure may be used:
  - o Berg Balance Scale (BBS) [11]
  - o Functional Ambulation Category (FAC) [12] – for the assessment of ambulation status by determining how much human support the patient requires when walking, regardless the use of personal assistive device.
  - o 6 min walk test (6MWT) [13]
- *Level of limbs strength or weakness (e.g. hemiparesis or hemiplegia),* and *preserved motor function.* In a prognostic view, these aspects can be assessed by testing key muscles with:
  - o Medical Research Council (MRC) [14]. The most important movements with prognostic value are shoulder abduction and finger extension (SAFE), and hip extension [15, 16]
  - o NIHSS [8] – subitem 5a, 5b, 6a, 6b
  - o Fugl-Meyer Assessment – Upper Extremity (FMA-UE) and Lower Extremity (FMA-LE) [17]
  - o Action Research Arm Test (ARAT) [18]
  - o Motricity Index (MI) [10]
- *Assessment of somatosensory function (e.g. light touch, pressure, stereognosis, pain, two-point discrimination) and coordination.* Sensation function is not used as a predictor of motor recovery. However, the most used outcome measure for it is the NIHSS [8] – sub-item 7, 8.
- *Reflex testing and muscle tone.* Despite these two aspects are important for the complete neurological examination, they are not included as predictors of motor recovery.

#### Steps 4 and 5. Application of prognostic tools and development of a plan of care

After complete collection of clinical information (i.e. clinical, motor, neurological, functional), the subsequent step is a synthesis and interpretation of main findings. Through examination and assessment, the process of establishing a therapeutic alliance with the patient and setting of rehabilitation goals is kicked-off. To this end, clinicians must consider patient’s goals for negotiating shared therapeutic goals and tailoring personalized rehabilitation interventions to what is meaningful and achievable. At this stage, prognosis can be considered as the expected degree of recovery as derived by prognostic tools. In case that prognostic tools are missing, clinicians can only focus the rehabilitation interventions on improving residual motor function, according to results from the initial assessment.

**Fig. 2:**
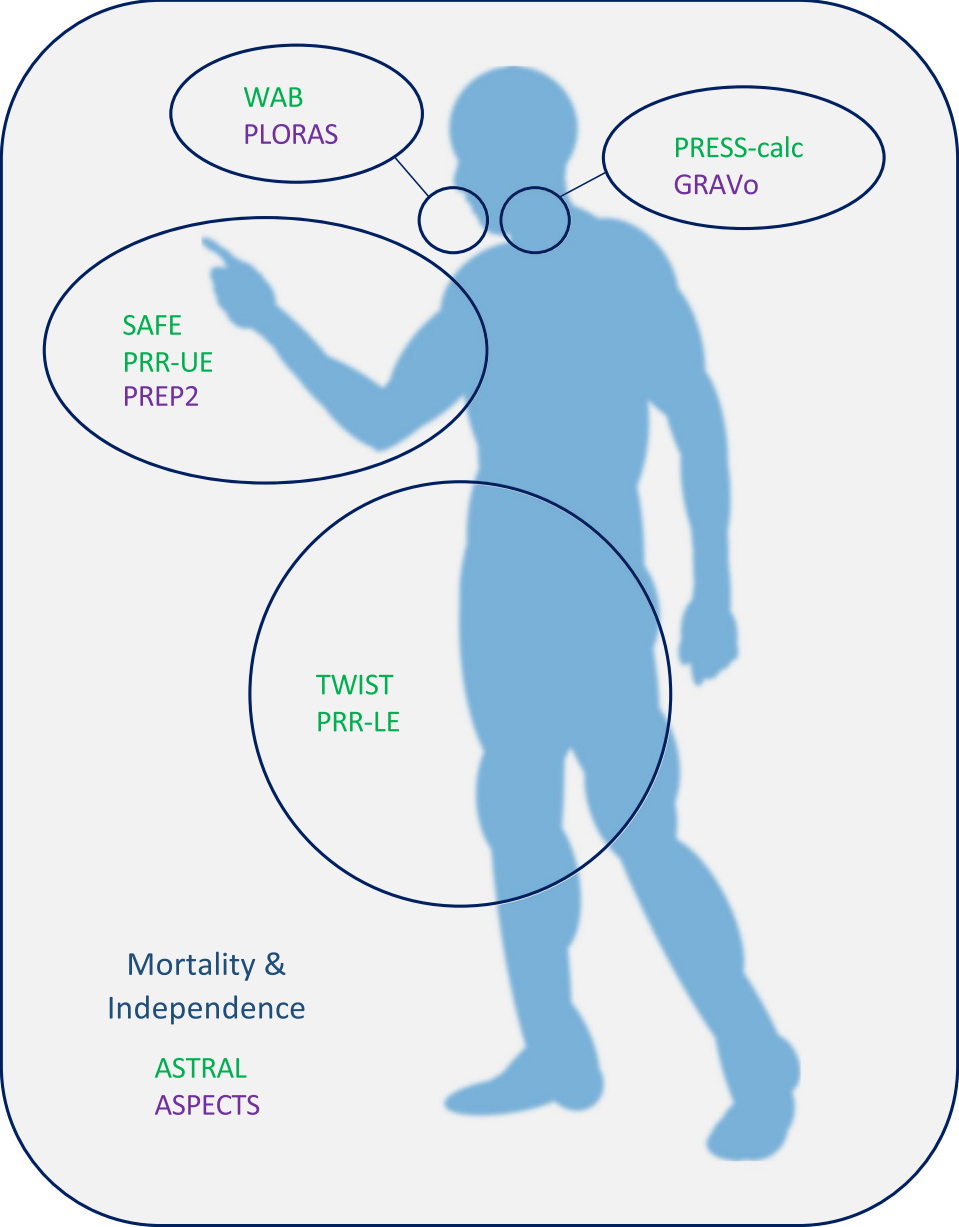
Schematics of the prediction tools available, according to the body region and function examined, and whether they require only clinical testing (green) or additional neurophysiological/neuroimaging evaluations (purple)

To understand which tools are available along the recovery process, we propose a synoptic table summarising prognostic tools applicable at different time points after stroke (Table 1). Furthermore, Fig. 2 depicts which tools are available to each body region and function, and whether they require only clinical examination or additional neuroimaging/neurophysiological testing.

**Table 1:** Prediction tools for recovery after stroke, at different time points

*Assessment time (baseline, T0)*	*Timing of predicted outcome (follow-up, T1)*							
	**15 days**	**30 days (1 month)**	**40 days**	**3 months**		**6 months**		**12 months**
**24 h (1 day)**				ASPECTS; ASTRAL *(Mortality & Independence)*				
**72 h (3 days)**	GRAVo *(PEG)*			PREP2 *(UE)*	WAB *(Language)*	PRR-UE; SAFE *(UE)*	PRR-LE *(LE)*	
**5 days**		*Language*		*Language*				*Language*
**7 days (1 week)**			PRESS calc *(Swallowing)*	TWIST *(LE)*		*UE*		
**10 days**						SAFE* (UE)*		
**30 days (4 weeks)**				*UE*				
**2–6 weeks**				*UE*				

*ASPECTS* Alberta Stroke Program Early Computed Tomography Score, *ASTRAL* Acute Stroke Registry and Analysis of Lausanne, *GRAVo* Glasgow Coma Scale, Race, Age, hematoma Volume, *LE* Lower Extremity, *PEG* Percutaneous Endoscopic Gastrectomy, *PREP2* Predict Recovery Potential, *PRESS* Predictive Swallowing Score, *PRR* Proportional Recovery Rule, *SAFE* Shoulder Abduction Finger Extension, *TWIST* Time to Walking Independently After Stroke, *UE* Upper Extremity, *WAB* Western Aphasia Battery

#### Prediction of placement of tube feeding and percutaneous endoscopic gastrostomy (PEG)

After haemorrhagic stroke, GRAVo tool (Table 2) is a clinical score for prediction of PEG placement during patient’s hospitalization [19]. Clinical information (i.e. Glasgow Coma Scale – GCS, race and age) is easily retrievable from first patient contact at admission, moreover intracerebral haemorrhage (ICH) volume is needed from a computed tomography (CT) scan.

**Table 2:** Description of GRAVo tool for prediction of PEG placement in hemorrhagic stroke

Assessment at 72 h	Parameter	GRAVo Points	Prediction at 15 days	Accuracy of prediction
GCS	GCS > 12	0	GRAVo ≥ 4 points PEG placement	Sensitivity = 58.62% Specificity = 84.73% AUC = 0.75
	GCS ≤ 12	2		
Race (African American)	no	0		
	yes	1		
Age	≤ 50 years	0	GRAVo ≥ 5 points PEG placement	Sensitivity 46.55% Specificity 93.13% AUC: n.a
	> 50 years	2		
ICH volume	ICH ≤ 30 cc	0		
	ICH > 30 cc	1		

*AUC* area under the curve, *GCS* Glasgow Coma Scale, *ICH* intracerebral haemorrhage, *n.a.* not available

#### Prediction of language function recovery

Predicting aphasia recovery after stroke is difficult, because of the influence of lesion, clinical features and treatment-related factors [20]. A 3 months-clinical prediction may be performed by knowing score from the Western Aphasia Battery (WAB) assessed at 72 h (Table 3) [21]. However, the most robust predictors of recovery seems to be lesion related factors; in particular some evidence suggest that circumscribed lesions in frontal, parietal or temporal lobes are related to good recovery at 1, 3 and 12 months, while extensive middle cerebral artery (MCA) disruption or extensive temporoparietal lesions are linked to persistent moderate or severe deficits at 1, 3 and 12 months [20, 22].

**Table 3:** Description of language function recovery at 3 months after stroke

Assessment at 72 h	Parameter	Prediction at 3 months	Accuracy of prediction	Note
WAB	WAB < 29 points	WAB _max_ – WAB _72 h_	Patient can recover 73% of maximal potential recovery	The role of treatment and its interference with recovery is not well understood

*WAB* Western Aphasia Battery

PLORAS (*predict language outcome and recovery after stroke)* is a repository of anatomical and functional imaging data of stroke patients (PLORAS Database), allowing prediction of the language function based on a single structural (anatomical, T1-weighted) brain scan. However, direct access to the data is password protected and limited to relevant members of the PLORAS Research team and local collaborators at University College London (UCL) [23].

#### Prediction of swallowing function recovery

For prediction of swallowing function after stroke, a prognostic model has never been validated. However, after dysphagic ischemic stroke, it is possible to use the online tool *Predictive Swallowing Score* (PRESS), for predicting functional oral intake at 40 days after stroke, according to clinical information (i.e. age, stroke severity, stroke location, risk of aspiration and impairment of oral intake) retrievable at 1 week after stroke [24, 25].

#### Prediction of mortality and independence level

After stroke, the global cognitive functioning measured with the Montreal Cognitive Assessment (MoCA), a validated screening tool ranging from 0 to 30 points (cognitive impairment defined as MoCA < 26 points), was the only predictive variable for return to work (RTW) [26, 27]. However, cut-off scores of MoCA for valid prediction of RTW are not reported. Alongside, for predicting mortality and independence level at 3 months after stroke, ASTRAL (Acute Stroke Registry and Analysis of Lausanne) and ASPECTS (*Alberta Stroke Program Early Computed Tomography Score*) scores have been developed according to clinical information collected at 24 h after stroke. ASTRAL score is an online calculator developed for mortality and independence level prognosis from 24 h to 3 months, or 5 years after ischemic stroke [28, 29]. The clinical information required at 24 h are age, severity of stroke (measured with NIHSS), stroke onset to admission time, range of visual fields, acute level of glucose and level of consciousness [28]. ASPECTS is a quantitative score evaluating lesion location in the MCA territory, based on CT scan of the hyperacute phase [30–32]. Ten brain regions are assigned either a score of 1 (normal) or 0 (ischaemic change), and the total sum score is calculated*.* Starting from a score of 10, 1 point is lost for each brain region involved. ASPECTS demonstrated a sensitivity of 0.78 and specificity of 0.96 for the prognosis of functional independence at three months based on the modified Rankin Scale, with a cut-off of 7 or lower clearly discriminant between functional independence and dependence or death, at three months (i.e. ASPECTS score < 7 predicts poor functional outcome). Similar results were obtained with the pc-ASPECTS scale, adapted to stroke in the posterior cerebral artery, where pons and midbrain are scored 2 points each [33].

#### Prediction of upper limb function recovery

Cortico-Spinal Tract (CST) is responsible for muscles activation and control, with a critical role for finger extensors [34]. It is widely acknowledged that presence of active SAFE is a reliable clinical sign predicting upper limb recovery at medium-long term after stroke [35]. SAFE movements could be present either at 72 h and within six weeks after stroke, allowing to predict active motor recovery at three or six months, with regard to ARAT or FMA-UE [16, 35–37]. Moreover, it was reported as the strongest predictor for bimanual performance [38]. Intactness of CST is better expressed by the presence of finger extension than shoulder abduction, thus the earlier it appears after stroke, the higher the probability of regaining arm motor function [37]. Alongside SAFE, other validated outcome measures (e.g. FMA-UE) were used to predict recovery of UL function [35, 39]. The *Proportional Recovery Rule* (PRR) [39], mainly based on the FMA-UE, showed that 70% of the patients recover approximately 70% of their maximal improvement potential (*recoverees*), while 30% of them do not (*non-recoverees*). The *non-recoverees* were defined as the patient with severe impairment at 72 h (i.e. Fugl-Meyer Lower Extremity < 18 points, 0 < FMA-UE < 17, facial palsy and no finger extension) [39]. This rule has been criticized for its statistical and mathematical methods, because of the confounding nature of the correlation between initial scores and change over time [40, 41]. Anyway, neither FMA-UE, nor SAFE have ever been investigated in prognostic models with baseline assessment performed later than six weeks after stroke, thus prediction of arm motor recovery can be performed with certain degrees of evidence only within this timeframe.

Finally, the presence of some of the following features have positive predictive value on UL prognosis, after stroke [42]:

- Sex (male)
- Preserved CST
- Stroke on the left hemisphere
- High UL function
- Low
  - o Age (the younger the better)
  - o Global disability
  - o UL and LL impairment
- Absence of
  - o urinary incontinence
  - o sensation deficit
  - o visual disorder
- Presence of
  - o motor evoked potential (MEPs). Whenever TMS or information on the presence of MEPs are not available, a clinically valid surrogate is the presence or absence of any visible muscle contraction when attempting to perform shoulder abduction and finger extension (SAFE) [43].
  - o somatosensory evoked potentials (SSEPs)

To date, the *Predict Recovery Potential* (PREP2) algorithm (Table 4) is the only validated predictive model for UL recovery, considering clinical and instrumental parameters to be collected within 72 h after stroke. It can predict arm recovery after 3 months according to ARAT, with an overall accuracy of 75%. This algorithm allows to categorize patients according to certain combinations of information such as age, SAFE strength, presence of motor evoked potentials (MEPs) in the motor cortex and level of neurological status (i.e. NIHSS). TMS has to be performed only in case of SAFE < 5, and then NIHSS only when MEPs are not present [16, 36].

**Table 4:** Description of the PREP2 algorithm, for prediction of UL recovery

Assessment at 72 h	Parameters cut-off	Prediction at 3 months	Accuracy of prediction
Age Strength (MRC) at SAFE TMS (MEPs) NIHSS	SAFE ≥ 8 and age < 80 y	Excellent (ARAT 50–57)	75%
	5 ≤ SAFE < 8 and age > 80 y	Good (ARAT 34–48)	
	SAFE < 5, MEP + and NIHSS < 7	Limited (ARAT 13–31)	
	SAFE < 5, MEP- and NIHSS ≥ 7	Poor (ARAT 0–9)	

*ARAT* Action Research Arm Test, *MEPs* Motor Evoked Potentials, *NIHSS* National Institute for Health Stroke Scale, *SAFE* Shoulder Abduction, Finger Extension, *TMS* Transcranial Magnetic Stimulation

#### Prediction of lower limb & walking function recovery

Recovery of walking activity depends on initial lower-limb motor impairment, stroke severity, trunk control and balance, age, lower-extremity (LE) sensory impairment, homonymous hemianopia or visuospatial inattention, presence or absence of motor-evoked potential elicited in tibialis anterior, lesion location and lesion overlap with the corticospinal tract [44]. As well as for UL, the PRR exists also for the LE, stating that patient after stroke can recover 64% of the difference between the total score of the FMA-LE (i.e. 34 points) and the initial score. From this model it seems that patients scoring FMA-LE ≥ 14 are 100% likely to follow the rule, while those scoring below 14 points are 35% likely to follow the rule [45]. Moreover, similar to the PREP2 algorithm for the UL, an algorithm for predicting recovery of walking ability has been developed [15]. Is called the *Time to Walk Independently after Stroke* (TWIST) algorithm and predicts the time taken to walk independently or not after stroke, according to Functional Ambulation Category (FAC). It requires an assessment at 1 week of strength hip extension (MRC) and trunk control function (TCT) (Table 5).

**Table 5:** Description of the TWIST algorithm, for prediction of walking recovery

Assessment at 1 week	Parameters cut-off	Prediction	Accuracy of prediction
TCT Hip extension (MRC)	TCT > 40	FAC > 3 at 6 weeks	91%
	TCT < 40 and MRC ≥ 3	FAC > 3 at 12 weeks	100%
	TCT < 10 and MRC < 3	Dependent at 12 weeks	100%

*FAC* Functional Ambulation Category, *MRC* Medical Research Council, *TCT* Trunk Control Test

### Biomarkers for prediction of motor recovery after stroke

As reported, motor recovery after stroke is associated with initial impairment and CST integrity. Clinical assessment is a strong independent predictor, especially for patients with mild to moderate impairment [39]. However, for severely impaired patients, prognostic models may benefit by the inclusion of neuroimaging and neurophysiology biomarkers [46]. This is critically important for the design of interventional trials early after stroke, where patient inclusion and stratification based on potential neurobiological recovery would greatly benefit from accurate prognostic models [46]. To this end, core recommendations have been recently established for biomarkers ready to be used in research clinical trials [46]. To sum up, the most important biomarkers are integrity of CST indexed by Diffusion Tensor Imaging (DTI) or by lesion overlap, and Transcranial Magnetic Stimulation (TMS) measure of Motor Evoked Potentials (MEPs) of the upper limb [46]. For the prediction of motor recovery after stroke, TMS has been used to investigate the functional integrity of the CST; patients where MEP + could be elicited were classified as having relatively preserved CST, whereas the absence of MEP + was indicative of severe disruption of CST integrity. In line with this hypothesis, when considering patients with initial severe upper limb motor impairment, those with MEP + showed higher recovery potential than those with MEP-. Both for DTI and TMS measure there is evidence of their moderate to strong relationship with baseline impairment add recovery potential [46]. Afterwards, measures of resting-state functional connectivity are still in a developmental status for the prediction of treatment response in all the stages of stroke recovery. However, for the identification of poor *recoverees* early after stroke the PREP2 should be considered whenever TMS assessment is available, while a clinically valid surrogate outcome is the presence/absence of any visible muscle contraction when attempting to perform SAFE [43].

### Online tools for assessment and monitoring of stroke recovery

Time constraints has been reported by clinicians as a major barrier to undertake assessment and individualized treatment planning based on the available evidence [47]. To overcome this issue and to assist the decision-making process there is growing interest towards tools providing useful information in a rapid and reliable way. Following, we summarize some online tools available both for prediction and a comprehensive assessment and treatment-decision making during daily clinical practice:

- PRESS calc: it is a smartphone application to predict recovery of functional oral intake from 1 week to 40 days after dysphagic stroke [24, 25].
  - o Apple iOS: https://apptopia.com/ios/app/1401176212/about
  - o Google Play: https://play.google.com/store/apps/details?id=ch.kssg.press
- ASTRAL score: to predict disability and death over 12 months and 5 years after acute ischemic stroke [28, 29].
  - o Online calculator available: https://www.mdcalc.com/astral-score-ischemic-stroke
- ViaTherapy: it is a smartphone validated application developed by healthcare institutions and clinicians with the goal of guiding therapists from assessment to treatment selection [48]. The tool serves as indication to select evidence-based treatments specific to patient’s stage of recovery and functional status.
  - o Apple iOS:https://apps.apple.com/us/app/viatherapy/id1108116302?ign-mpt=uo%3D4
  - o Google Play: https://play.google.com/store/apps/details?id=org.viatherapy.androidapp
- Dynamic prediction of Vliet et al. 2020 [49] consists in a user-friendly online platform for 5 strata classification of patients recovery, based on FMA-UE assessment (https://emcbiostatistics.shinyapps.io/LongitudinalMixtureModelFMUE/). Taken together, ViaTherapy and dynamic predictions allows clinicians to access evidence-based tools for assessment, prognosis, and treatment selection.
- Rehabilitation Measure Database: https://www.sralab.org/rehabilitation-measures. It is a database where to find more than 500 rehabilitation outcome measures with instrumental details for each of them.
- Outcome Measures Recommendations: https://www.neuropt.org/practice-resources/neurology-section-outcome-measures-recommendations. It is a database of recommendations for outcome measures used in clinical practice and research of the main neurological diseases (i.e. Multiple Sclerosis, Stroke, Traumatic Brain Injury, Parkinson Disease, Vestibular Disorders, Spinal Cord Injury).
- Assessment of Life Habits (LIFE-H): https://strokengine.ca/en/assessments/assessment-of-life-habits-life-h/. It is an outcome measure to assess the quality of social participation of people with disability by estimating how the patient accomplishes ADLs and social roles. It is worth noticing because of its nature of being an outcome measure for the Participation domain of International Classification of Functioning, Disability and Health (ICF).
- Stroke Rehabilitation Clinician Handbook: http://www.ebrsr.com/sites/default/files/EBRSR%20Handbook%20Chapter%204_Upper%20Extremity%20Post%20Stroke_ML.pdf. It is a book for the clinical management of UL after stroke.
- Evidence-Based Review of Stroke Rehabilitation: www.ebrsr.com. It is a portal where to find the most updated evidence of the clinical management of stroke rehabilitation.

## Conclusion

In this paper we have summarized the state of the art on tools available for prognosis of recovery after stroke (i.e. PRESS calc, GRAVo, PREP2, ASTRAL score) [16, 19, 24, 25, 28]. To date, long-term prediction accuracy and impact of use in clinical care has been established only for PREP2, which is symptomatic of the yet undeveloped stage of validation and implementation for prognostic tools [36]. To facilitate the adoption of prognostic tools, we proposed a clinically-oriented organization of available knowledge previously missing in the literature. The aims were to provide clinicians with a stepwise approach for patient’s evaluation, to improve confidence on planning a personalized neurorehabilitation program, with a prognostic perspective. Implications for clinical practice may be summarized as follows:

- In the field of neurorehabilitation, several prognostic tools have been proposed and are ready to be included within a systematic patient’s evaluation.
- Prognostic tools are useful for patients and clinicians in many ways: set meaningful and achievable goals, design personalized interventions, and interpret recovery outcomes.
- A neurorehabilitation intervention can no longer be defined optimal, if neglecting the use of available prediction models. Validated tools are cooperative, not competitive, to clinician’s judgement, and should be embraced as an objective guide in the decision-making process.

However, for all the prognostic tools here reported, statistical accuracy and power have not been validated yet, therefore the reliability of their use needs to be balanced by clinical judgement.

We suggest clinicians to reconsider the importance of performing a proper assessment as part of the rehabilitation program.

Moreover, we strongly suggest researchers in neurorehabilitation to strive the concept of prediction rather than prognosis, by developing prediction model that considers rehabilitation interventions as a putative factor for motor recovery.

## Data Availability

Not applicable.

## Abbreviations

ARAT: Action Research Arm Test
ASPECTS: Alberta Stroke Program Early Computed Tomography Score
ASTRAL: Acute Stroke Registry and Analysis of Lausanne
BBS: Berg Balance Scale
CST: Corticospinal Tract
CT: Computed Tomography
DTI: Diffusion Tensor Imaging
EEG: Electroencephalogram
EMG: Electromyography
FAC: Functional Ambulation Categories
FMA-LE: Fugl-Meyer Assessment Lower Extremity
FMA-UE: Fugl-Meyer Assessment Upper Extremity
GCS: Glasgow Coma Scale
GRAVo: Glasgow Coma Scale, Race, Age, hematoma Volume
ICF: International Classification of Functioning, Disability and Health
LE: Lower extremity
MCA: Middle cerebral artery
MEP: Motor evoked potentials
MI: Motricity index
MoCA: Montreal Cognitive Assessment
MRC: Medical research council
MRI: Magnetic resonance imaging
NIHSS: National Institutes of Health Stroke Scale
PEG: Percutaneous Endoscopic Gastrectomy
PLORAS: Predict Language Outcome and Recovery After Stroke
PRESS: Predictive Swallowing Score
PRR: Proportional Recovery Rule
RTW: Return to Work
SAFE: Shoulder Abduction Finger Extension
6MWT: 6 Minutes walking test
SSEP: Somatosensory evoked potentials
TCT: Trunk Control Test
TMS: Transcranial Magnetic Stimulation
TWIST: Time to Walking Independently After Stroke
WAB: Western Aphasia Battery
UCL: University College London
UL: Upper Limb
